# Ruthenium-Alloyed Iron Phosphide Single Crystal with Increased Fermi Level for Efficient Hydrogen Evolution

**DOI:** 10.1021/acsami.2c16419

**Published:** 2022-12-09

**Authors:** Yu Kang, Yujia Han, Hedong Chen, Horst Borrmann, Peter Adler, Darius Pohl, Martin Hantusch, Markus König, Yangkun He, Yufei Ma, Xiaodong Wang, Claudia Felser

**Affiliations:** † Max Planck Institute for Chemical Physics of Solids, Nöthnitzer Straße 40, 01187 Dresden, Germany; ‡ Dalian Institute of Chemical Physics, Chinese Academy of Sciences, 457 Zhongshan Road, Dalian 116023, China; § Dresden Center for Nanoanalysis, cfaed, Technische Universität Dresden, Helmholtzstraße 18, 01069 Dresden, Germany; ∥ Leibniz-Institute for Solid State and Materials Research (IFW), Dresden 01069, Germany; ⊥ School of Materials Science and Engineering, Beihang University, Beijing 100191, China

**Keywords:** iron phosphide alloy, ruthenium, single crystal, electronic structure, hydrogen evolution

## Abstract

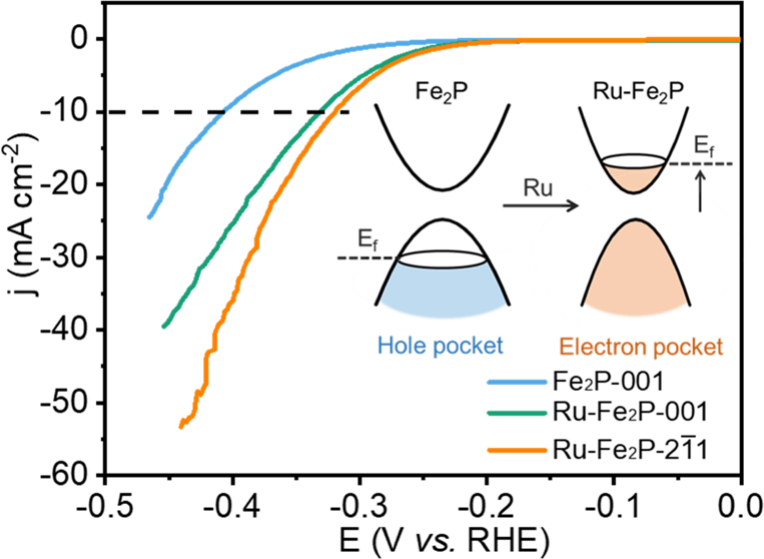

Transition metal phosphide alloying is an effective approach for optimizing the electronic structure and improving the intrinsic performance of the hydrogen evolution reaction (HER). However, obtaining 3d transition metal phosphides alloyed with noble metals is still a challenge owing to their difference in electronegativity, and the influence of their electronic structure modulated by noble metals on the HER reaction also remains unclear. In this study, we successfully incorporated Ru into an Fe_2_P single crystal via the Bridgeman method and used it as a model catalyst, which effectively promoted HER. Hall transport measurements combined with first-principles calculations revealed that Ru acted as an electron dopant in the structure and increased the Fermi level, leading to a decreased water dissociation barrier and an improved electron-transfer Volmer step at low overpotentials. Additionally, the (21̅1) facet of Ru–Fe_2_P was found to be more active than its (001) facet, mainly due to the lower H desorption barrier at high overpotentials. The synergistic effect of Ru and Fe sites was also revealed to facilitate H* and OH* desorption compared with Fe_2_P. Therefore, this study elucidates the boosting effect of Ru-alloyed iron phosphides and offers new understanding about the relationship between their electronic structure and HER performance.

## 1 Introduction

Electrochemical water splitting for high-purity hydrogen production has been considered one of the most promising methods for storing the energy harvested from renewable energy resources such as wind and sunlight.^1−5^ Currently, platinum-based materials are the benchmark electrocatalysts for the hydrogen evolution reaction (HER) because of their favorable H adsorption ability; however, their scalable commercial applications are greatly limited by the scarcity and high cost of noble metals.^6,7^ Many studies have been devoted to decreasing the Pt loading on the electrode or developing noble-metal-free catalysts for the HER.^8−10^ Transition metal phosphides have emerged as competitive electrocatalysts owing to their abundant reserves, low cost, and high conductivity.^11−14^ Phosphides have a rich variety of compounds because their metal types and phosphorus contents can be extensively tuned, allowing the optimization of the catalytic electronic structure.^15,16^ Interestingly, the metal atoms in many phosphide structures still maintain their metallic state with a near (0) valence state, probably due to the extensive metal–metal bonding network that diminishes the degree of metal-to-phosphorus electron transfer,^17^ which is favorable for the HER. To date, many phosphides have been explored, including the most popular 3d transition metal phosphides, such as MnP*_x_*, FeP*_x_*, CoP*_x_*, and NiP*_x_*.^18−22^ In addition, phosphides with different phosphorus contents in the structure have been studied. For example, Fe_3_P, Fe_2_P, FeP, and FeP_2_ can be used for water splitting, and they exhibit different HER activities.^16,23^ However, monomeric phosphides usually exhibit low intrinsic reactivity. Therefore, further tailoring of the electronic structure is highly desirable for optimal HER performance.

Inspired by metal alloys with promoted reactivity, alloying transition metals to form binary or ternary phosphides could be an effective strategy to improve their performance.^24−27^ The metals in these alloys have different valence electrons, and their band structures may be modified. For instance, Lou et al. synthesized Ni-doped FeP nanocrystals, which outperformed the monomeric FeP structure for HER owing to the synergistic modulation of the component and the electronic properties of the Ni dopant.^28^ Many other bimetallic materials such as Ni–Co–P, Fe–Co–P, V–CoP, and Mn–Co–P have also been reported.^29−32^ These alloys with abundant active sites can modify the electronic configuration and consequently optimize the hydrogen binding on the surface. Here, it can be seen that most of the alloys are combinations of 3d transition metal (e.g., Fe, Co, Ni, Mn) phosphides. However, their reactivities are not as competitive as those of noble-metal-based electrocatalysts.

A small amount of noble metals incorporated into phosphides to form alloys can be a favorable solution to further improve the HER performance and maintain the balance of good cost-competitiveness. However, obtaining 3d transition metal phosphides alloyed with noble metals is challenging, and only a few studies have been reported to date.^33−35^ This was probably due to the preferential phosphorization of 3d metals over noble metals. Furthermore, uniform distributions of the alloy compositions and phosphorus contents are difficult to achieve using conventional methods.^30,36,37^ As a result, the influence of noble metals on the electronic structure and HER mechanism remain unclear.

To address these issues, the Bridgeman method was used to melt Ru and Fe phosphides to generate a homogeneous phase. Ru-incorporated Fe_2_P single crystals were successfully synthesized by slow cooling, resulting in a uniform distribution of Ru in Fe_2_P. Electrochemical tests revealed that Ru-alloyed Fe_2_P could significantly improve the HER kinetics compared to pure Fe_2_P, indicating the effectiveness of the alloying method. In addition, the Ru–Fe_2_P single crystal can be used as a model catalyst to study the crystalline anisotropy of the surface reactivity, and its (21̅1) and (001) planes were compared in the HER. Density functional theory (DFT) calculations were conducted to differentiate the HERs, reveal the electronic structure, and elucidate reaction mechanisms. Thus, this study may facilitate the design of noble-metal-alloyed phosphide catalysts with high intrinsic reactivity.

## 2 Results and Discussion

### 2.1 Crystal Structure and Transport Measurements

**Figure 1:**
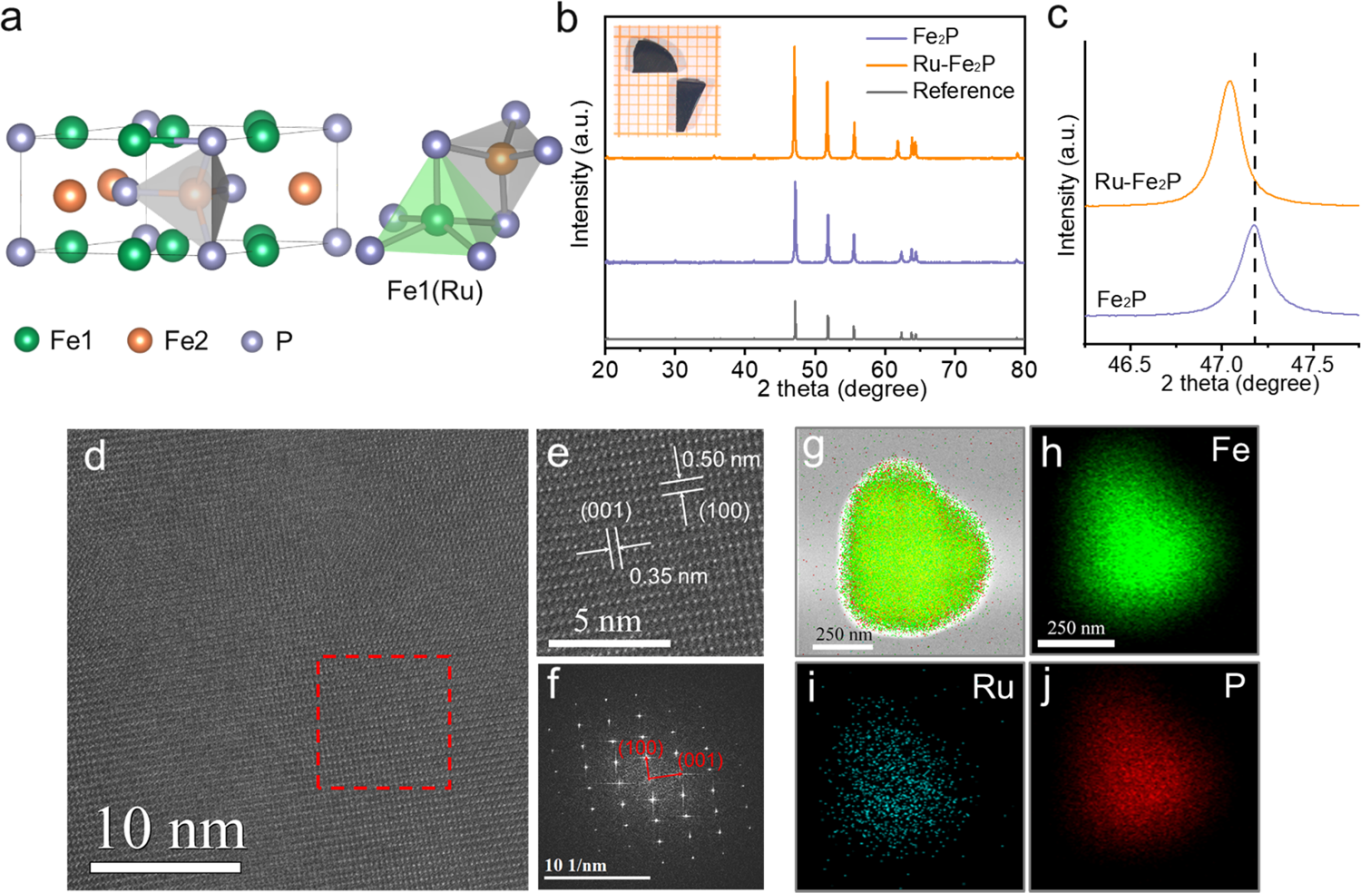
Structural characterizations of Fe_2_P and Ru–Fe_2_P. (a) Crystal structure illustration of Fe_2_P, in which Fe atoms occupy the tetrahedral and pyramidal sites via P coordination. (b) Powder XRD patterns of the two samples (the inset is the photograph of the single crystals) and (c) the magnified XRD patterns. The reference pattern is derived from the simulation of Fe_2_P standard CIF file. (d, e) HRTEM images of the Ru–Fe_2_P single crystal and (f) the corresponding FFT image. (g–j) Elemental mapping results from energy-dispersive X-ray spectroscopy.

We successfully grew large-sized Fe_2_P and Ru–Fe_2_P single crystals using the Bridgeman method, in which Fe_2_P crystallized in the noncentrosymmetric hexagonal space group *P*6̅2*m* (189). As shown in Figure 1a, the space group combines two inequivalent Fe sites in its structure, that is, pyramidal (Fe1) and tetrahedral (Fe2) sites coordinated by P atoms. Furthermore, Ru atoms can be incorporated into the structure. Here, the composition of Ru–Fe_2_P was determined to be (Fe_0.965_Ru_0.035_)_2_P by inductively coupled plasma (ICP) atomic emission spectrometry. We tried to increase the Ru amount to the component of (Fe_0.93_Ru_0.07_)_2_P, but we failed to obtain the single crystal. Thus, only (Fe_0.965_Ru_0.035_)_2_P crystal is discussed in the following part. Subsequent powder X-ray diffraction (XRD) patterns in Figure 1b show that the samples exhibit a hexagonal structure, with the inset showing photographs of the well-crystallized samples. The main peak of Fe_2_P at approximately 47.2° shifts to a lower scattering angle after Ru incorporation (Figure 1c), in agreement with the fact that Ru has a larger atomic radius than Fe, which leads to lattice expansion in the structure. The crystal structure of Ru–Fe_2_P was further confirmed using high-resolution transmission electron microscopy (HRTEM), and the results show good crystalline quality (Figure 1d,e). Two different lattice distances of 0.35 and 0.50 nm were observed, corresponding to the (001) and (100) planes of the hexagonal Ru–Fe_2_P.^38^ The structure and planes were further confirmed by fast Fourier transform (FFT, Figure 1f). In addition, the elemental mapping results indicate the homogeneous distribution of Ru, Fe, and P in the sample (Figure 1g–j).

The valence states of the metal atoms in Ru–Fe_2_P were identified by X-ray photoemission spectroscopy (XPS). As shown in Figure S1a, the Ru 3p spectrum was deconvoluted into two peaks. The small peak at 463.8 eV can be ascribed to the RuO*_x_* species,^39,40^ and the main peak at 461.5 eV is attributed to the Ru–P species.^41^ Notably, this binding energy is approximately equal to that of Ru(0).^39^ Meanwhile, the deconvoluted Fe–P binding energy of 707.1 eV in the Fe 2p spectra (Figure S1b) is also close to that of Fe metal.^16,17,42^ These results indicate that both the Ru and Fe atoms in Fe_2_P are in a metallic state, which is favorable for the HER.

To elucidate the incorporation of Ru atoms in the Fe_2_P structure, single-crystal XRD measurements and refinements were performed. Tables S1 and S2 show the refinement results and the detailed crystal parameters, respectively. Ru atoms occupy the 3f Wyckoff position, which is the same site as that of Fe1, suggesting that Ru substitutes the Fe1 site. This is because the Fe–P distance (Table S2) in the pyramidal environment (Fe1) is longer than that in the tetrahedral environment (Fe2); therefore, Ru, with a larger atomic radius, would favorably occupy the Fe1 site that has a larger space. In addition, the value of Ru content derived from the refinement result ((Fe_0.97_Ru_0.03_)_2_P) is very similar to the actual result obtained by ICP, indicating good refinement of the sample. The substitution was also studied by ^57^Fe Mössbauer spectroscopy in the magnetically ordered phase at low temperature (6 K). As shown in Figure S2, a strong broadening of the spectrum compared to that of Fe_2_P was observed after Ru substitution. This reflects the fact that the magnetic exchange network is strongly disturbed by the nonmagnetic Ru atoms. The Fe1 site signal with a larger hyperfine field splits into two subspectra, suggesting that the surrounding coordination environment has changed because of the Ru substitution. Also, the Fe2 site signal splits as the Fe2 exchange interactions are influenced by a Ru atom in its environment. These results are consistent with those from the XRD analysis.

**Figure 2:**
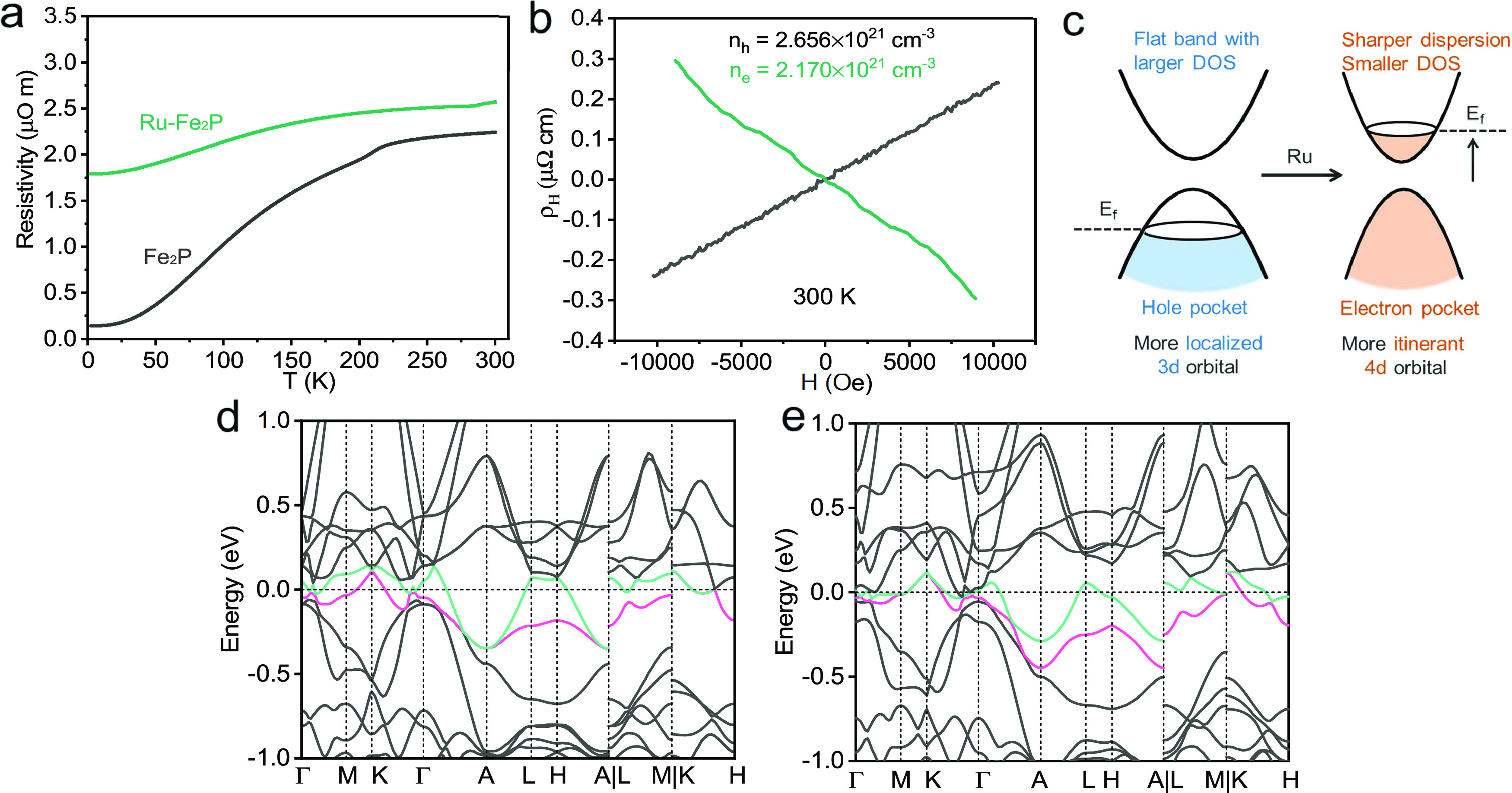
Transport measurements and band structures of Fe_2_P and Ru–Fe_2_P. (a) Plot of resistivity vs temperature and (b) Hall transport measurements of the single crystals. (c) Illustration of the charge carrier types. Band structures of (d) Fe_2_P and (e) Ru–Fe_2_P from the DFT calculations.

Transport measurements that reflect some parts of the electronic structure were also conducted. As shown in Figure 2a, the resistivity of the two single crystals increased with increasing temperature, demonstrating their metallic state.^43,44^ Owing to the enhanced lattice scattering of electrons derived from Ru incorporation in the structure, the resistivity of Fe_2_P was lower than that of Ru–Fe_2_P. At room temperature, Fe_2_P and Ru–Fe_2_P exhibited resistivities of 2.24 and 2.57 μΩ·m, respectively, which indicate that they are good conductors for electrochemical processes. Figure 2b presents the Hall resistivity for Fe_2_P and Ru–Fe_2_P. One can clearly observe the opposite magnetic field dependence of the Hall resistivity. This can be attributed to the different charge carrier types for the two crystals.^45,46^ The main charge carriers in Fe_2_P are holes with a density of 2.656 × 10^21^ cm^–3^, whereas the main charge carriers in Ru–Fe_2_P are electrons with a density of 2.170 × 10^21^ cm^–3^. Therefore, the incorporation of Ru into the structure induced a charge carrier-type transition. Figure 2c shows the band structure illustration of the two samples. The valence band of Fe_2_P is not fully filled by electrons and forms the “hole pocket” on top of the band, which dominates the transport measurement. In addition, 4d orbitals of Ru are more itinerant than the 3d orbitals of Fe, and the energy distribution is more dispersive, leading to a smaller density of states (DOS) and higher occupation level under the same electron amounts. As a result, the bottom state of the Ru–Fe_2_P valence band is partially occupied by electrons, and the “electron pocket” acts as the charge carrier.^47^ Consequently, the Fermi level increases after the incorporation of Ru. To further demonstrate the band structure, first-principles DFT calculations were conducted, and the results are shown in Figure 2d,e. Generally, the band structures of Fe_2_P and Ru–Fe_2_P (3.5% Ru) are similar. One can compare the red and green lines that are near the Fermi level. For Ru–Fe_2_P, both the red and green bands shift down relative to the Fermi level, particularly at Γ–A and L–H, in comparison to those of Fe_2_P. This indicates that more bands with higher energies can be occupied by electrons, which leads to an increase of the Fermi level. Therefore, these results suggest that a small amount of Ru can act as an electron dopant in the structure and promote the Fermi level.

### 2.2 Electrochemical Measurements

**Figure 3:**
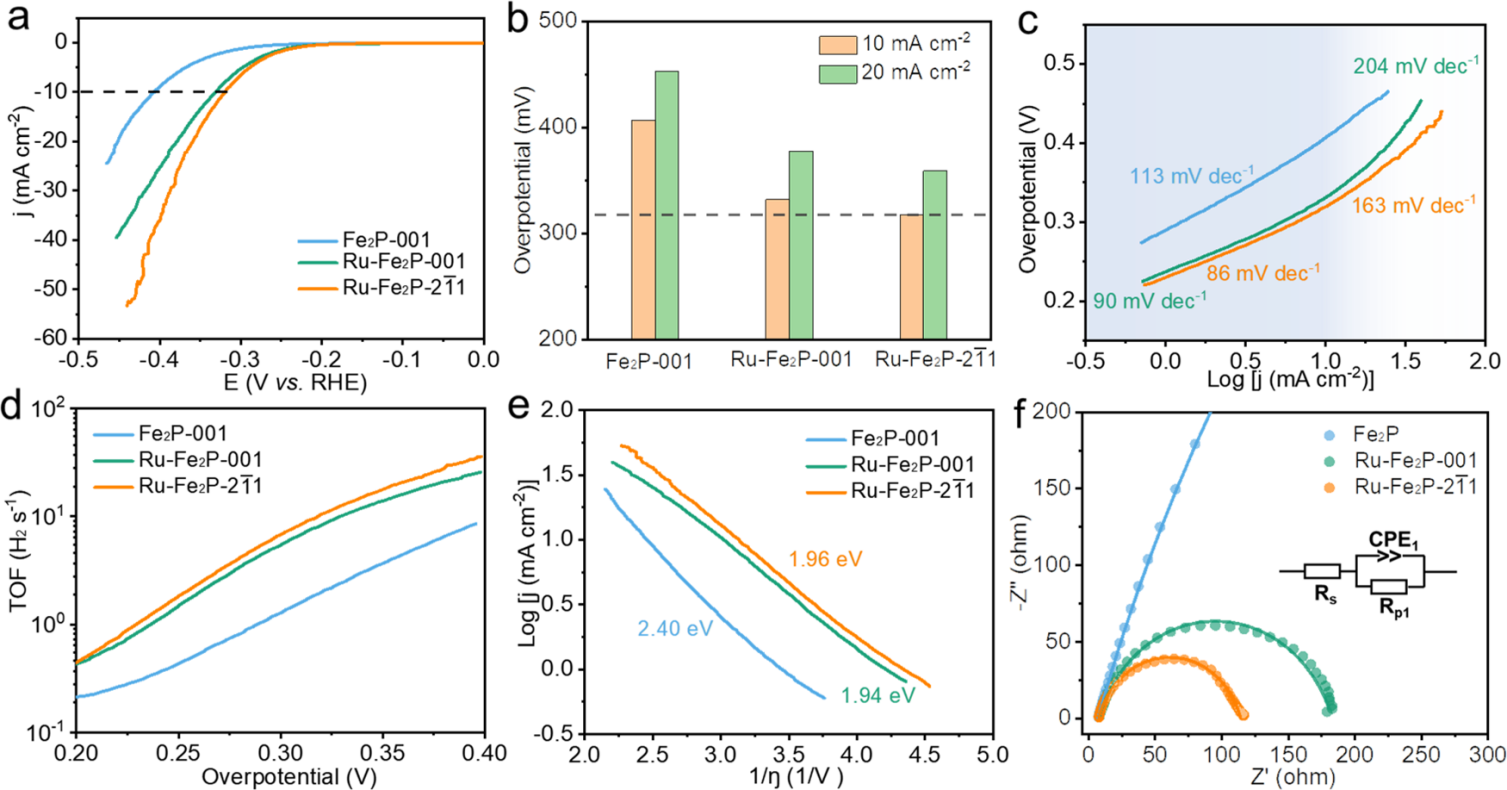
Electrochemical reactivities of Fe_2_P and Ru–Fe_2_P. (a) Linear sweep voltammetry curves for the single crystals at a scan rate of 2 mV s^–1^ and (b) the corresponding overpotentials at 10 and 20 mA cm^–2^. (c) Tafel slopes and (d) TOF results. (e) Electrochemical activation energy and (f) Nyquist plots at the overpotential of 250 mV (the inset is the equivalent circuit for the EIS measurement).

Prior to electrochemical measurements, Fe_2_P and Ru–Fe_2_P single crystals were first orientated by Laue diffraction to expose their specific crystallographic planes (Figure S3). HER performance was then studied in 1 M KOH electrolyte. Figure 3a shows the polarization curves normalized to the geometric area. The current density of Fe_2_P with the (001) facet (denoted as Fe_2_P-001) was much lower than that of Ru–Fe_2_P with the (001) facet (Ru–Fe_2_P-001). Interestingly, the (21̅1) plane of Ru–Fe_2_P (Ru–Fe_2_P-21̅1) outperformed the (001) plane, indicating the occurrence of crystalline anisotropic reactivity. The overpotentials of Fe_2_P-001 at 10 and 20 mA cm^–2^ were 407 and 453 mV, respectively (Figure 3b). However, the overpotentials decreased to 332 and 378 mV for Ru–Fe_2_P-001 and 318 and 359 mV for Ru–Fe_2_P-21̅1.

To investigate the HER kinetics, Tafel slopes were analyzed, as shown in Figure 3c. The plots were overpotential-dependent and could be divided into two parts. Fe_2_P has a slope of 113 mV dec^–1^ at a low overpotential, which is approximately 120 mV dec^–1^, indicating that the Volmer step (H_2_O + e^–^ + M = M–H + OH^−^) might be the rate-determining step.^48,49^ The (001) and (21̅1) facets of Ru–Fe_2_P showed lower values of Tafel slopes of approximately 90 and 86 mV dec^–1^, respectively, suggesting that the Volmer step occurs faster than that in Fe_2_P. At a higher overpotential, the Tafel slopes of Ru–Fe_2_P-001 and Ru–Fe_2_P-21̅1 increased to 204 and 163 mV dec^–1^, respectively. Because the Volmer step is an electron transfer process, it would not be a rate-determining step at higher overpotentials. In contrast, the Tafel step (2M–H = H_2_ + 2M) is not associated with electron transfer and, therefore, may determine the reaction rate at higher overpotentials. These results were also consistent with those obtained using kinetic simulations in a previous study.^49^Table S3 compared Ru–Fe_2_P with other metal phosphides. The low apparent reactivity of the single crystal is mainly due to the low surface area reflected by the low double-layer capacitance (*C*_dl_), while the Tafel slope value is comparable to those of nanocatalysts, indicating a relatively fast kinetics. Overall, Ru incorporation could effectively promote HER performance in the Fe_2_P structure, and the reactivity could be tuned by exposing the specific plane.

Turnover frequencies (TOFs) of the single crystals were also calculated to evaluate the HER activity. As shown in Figure 3d, the TOF value of Fe_2_P is much lower than that of Ru–Fe_2_P in the whole overpotential area. For example, at the overpotential of 300 mV, Ru–Fe_2_P-21̅1 shows a TOF value of 6.91 s^–1^, which is higher than that of Ru–Fe_2_P-001 (5.46 s^–1^) and approximately 5 times higher than that of Fe_2_P-001 (1.30 s^–1^). In addition, it was possible to determine the electrochemical activation energy using a different method. For the typical Arrhenius equation, *k* = *k*_0_e^–E_a_/RT^, temperature is the driving force for the reaction to proceed. Here, the overpotential played the same role as the temperature in driving the reaction. Thus, the deformed equation can be written as *k* = *k*_0_e^–E_a_/nFη^, where *n* is the electron transfer number, *F* is the Faraday constant, and η is the overpotential. The derived results are shown in Figure 3e. Ru–Fe_2_P-21̅1 and Ru–Fe_2_P-001 exhibit similar electrochemical activation energies of 1.96 eV (189 kJ mol^–1^) and 1.94 eV (187 kJ mol^–1^), respectively, which is approximately 20% lower than that of Fe_2_P-001 (2.40 eV or 231 kJ mol^–1^), demonstrating the vital role of Ru in activating water splitting. Here, the electrochemical activation energy can be a good parameter for measuring catalyst reactivity. Electrochemical impedance spectroscopy (EIS) was also performed at an overpotential of 250 mV to explore the electron transfer process. As illustrated in Figure 3f, the spectra exhibiting one semicircle for the three samples can be fitted by the inserted circuit, with the corresponding parameters listed in Table S4. The charge transfer resistances of Ru–Fe_2_P-21̅1 and Ru–Fe_2_P-001 were 107 and 175 Ω, respectively, which were much lower than that of Fe_2_P (2270 Ω). These results are also consistent with the HER performance; thus, Ru incorporation facilitates fast electron transfer kinetics.

### 2.3 Theoretical Analysis

**Figure 4:**
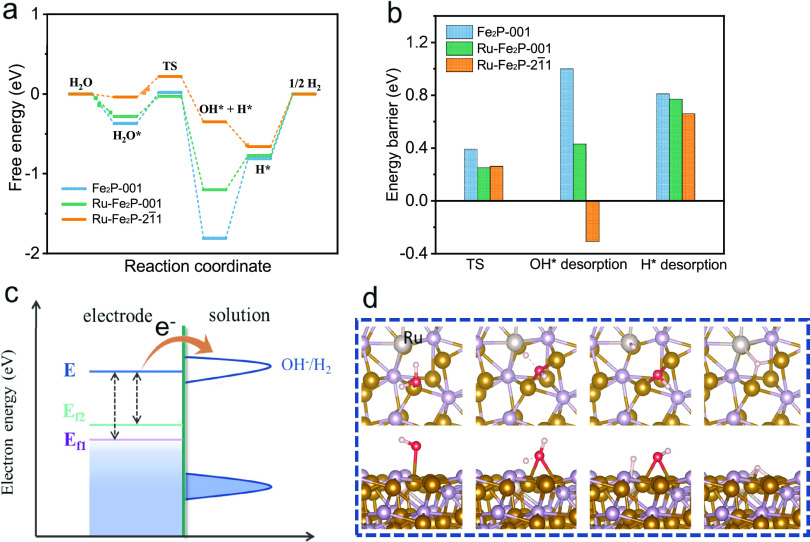
(a) HER path mechanisms and (b) the corresponding energy barriers for the three single crystals. (c) Influence of the Fermi level energy on electron transfer Volmer process during HER. (d) Active sites of reaction paths for the (21̅1) plane of Ru–Fe_2_P. The brown, purple, gray, red, and white balls represent the Fe, P, Ru, O, and H atoms, respectively.

To clarify the reasons for the reactivity difference among the three crystals, DFT calculations were carried out. As shown in Figure 4a, the reaction pathways involve several key steps, including H_2_O dissociation via a transition state, OH* desorption, and H* desorption forming H_2_. The three main energy barriers are summarized in Figure 4b. For the water dissociation step, the transition state barrier of Fe_2_P-001 was 0.39 eV, which was higher than those of Ru–Fe_2_P-001 (0.25 eV) and Ru–Fe_2_P-21̅1 (0.26 eV). Ru incorporation effectively reduced the dissociation barriers, which may have contributed to the HER performance. In the OH* desorption step with electron transfer, the barrier of Fe_2_P-001 (1.0 eV) was much higher than those of Ru–Fe_2_P-001 (0.43 eV) and Ru–Fe_2_P-21̅1 (−0.31 eV). The difficult desorption of OH* from Fe_2_P can result in the occupation of active sites and inhibition of kinetics, while Ru incorporation promoted this process, which is consistent with their Tafel slope results (113 vs 90 and 86 mV dec^–1^) at low overpotentials. Thus, the Volmer process (water dissociation followed by OH* desorption) is the rate-determining step for Fe_2_P. Figure 4c provides a brief illustration that explains how the Ru incorporation could improve the Volmer step in terms of electronic structure. The HER can proceed only if the electrochemical potential of the electrode is greater than the OH^–^/H_2_ potential, allowing the transfer of electrons to the unoccupied state to form H_2_. The incorporation of Ru, as an electron dopant, into the Fe_2_P structure increased the Fermi level. Thus, less electrostatic potential is required in the Ru–Fe_2_P to improve the Fermi level (*E*_f2_) energy to the electrochemical potential (*E*) for the HER compared with Fe_2_P (*E*_f1_), resulting in a promoted electron-transfer Volmer step. Finally, for H* desorption process, the energy barrier of Ru–Fe_2_P-21̅1 was approximately 0.66 eV, which was lower than that of Ru–Fe_2_P-001 (0.77 eV). This demonstrates that H_2_ generation was more favorable on the (21̅1) plane, and the Tafel step would be responsible for the crystalline anisotropy on surface reactivity of Ru–Fe_2_P at high overpotentials since the Volmer step is pre-equilibrated. Overall, the incorporation of Ru leads to an increased Fermi level, thus improving the Volmer and H desorption Tafel step. It should also be noted that the theoretical energy barriers here are slightly different from the electrochemical activation energy discussed above, probably due to the influence of real reaction kinetics, for example, diffusion.

The detailed active sites for the (21̅1) plane of Ru–Fe_2_P have been revealed in Figure 4d. H_2_O was dissociated on the Fe site, where the electronic structure has been tuned by near Ru atom. After dissociation, OH* desorption from the Fe site can be easily achieved with negative energy barrier thanks to the modulation of near Ru atom. Meanwhile, the H atom moved to the top hollow site of the Ru–Fe–Fe triangle, leading to a more favorable H* adsorption energy compared with Fe_2_P, which facilitated the desorption and H_2_ formation. Therefore, the synergistic effect of Ru on the Fe site plays a significant role in the elementary steps of water splitting.

## 3 Conclusions

In summary, we have successfully incorporated noble metal Ru into Fe_2_P single crystal using the Bridgeman method, which showed a much higher HER performance than pure Fe_2_P. Ru incorporation induced a transition of hole-type charge carriers into electron-type carriers, resulting in an increased Fermi level, which was demonstrated to facilitate the Volmer step, as illustrated by the different Tafel slopes of Fe_2_P and Ru–Fe_2_P at low overpotentials. Besides, the Ru–Fe_2_P single crystal, as a model catalyst, presented the crystalline anisotropy of the reactivity on different planes because of the Tafel step barrier differences at high overpotentials. Both Ru and Fe on the (21̅1) plane synergistically acted as the active sites during HER. This work not only offers a different synthesis approach for noble-metal-alloyed phosphides but also reveals the effect of electronic structure on HER after Ru incorporation, which may advance further designs of phosphide catalysts for efficient water splitting.

## Supporting Information Available

XPS spectra (Figure S1); Mössbauer spectra (Figure S2); Laue diffraction (Figure S3); top and side view of optimized, water dissociation (Figure S4 and S5); occupation states of atoms derived from the single crystal XRD refinement (Table S1); crystal data and parameters of the refinement (Table S2); comparison of metal phosphides (Table S3); electrochemical impedance spectra fitting parameters (Table S4) (PDF)

## References

[ref1] Li C.-F.; Zhao J.-W.; Xie L.-J.; Wu J.-Q.; Li G.-R. Fe Doping and Oxygen Vacancy Modulated Fe-ient Overall Water Splitting. Appl. Catal., B 2021, 291, 119987 10.1016/j.apcatb.2021.119987.

[ref2] Mohammed-Ibrahim J.; Sun X. Recent Progress on Earth Abundant Electrocatalysts for Hydrogen Evolution Reaction (HER) in Alkaline Medium to Achieve Efficient Water Splitting–a Review. J. Energy Chem. 2019, 34, 111–160. 10.1016/j.jechem.2018.09.016.

[ref3] Song D.; Sun J.; Sun L.; Zhai S.; Ho G. W.; Wu H.; Deng W. Q. Acidic Media Regulated Hierarchical Cobalt Compounds with Phosphorous Doping as Water Splitting Electrocatalysts. 2021, 11, 2100358. 10.1002/aenm.202100358.

[ref4] Wang Y. Z.; Yang M.; Ding Y. M.; Li N. W.; Yu L. Recent Advances in Complex Hollow Electrocatalysts for Water Splitting. Adv. Funct. Mater. 2022, 32, 2108681 10.1002/adfm.202108681.

[ref5] Kang Y.; He Y.; Pohl D.; Rellinghaus B.; Chen D.; Schmidt M.; Süß V.; Mu Q.; Li F.; Yang Q.; et al. Identification of Interface Structure for a Topological CoS_2_ Single Crystal in Oxygen Evolution Reaction with High Intrinsic Reactivity. ACS Appl. Mater. Interfaces 2022, 14, 19324–19331. 10.1021/acsami.1c24966. 35468289 PMC9073842

[ref6] Li C.; Baek J.-B. Recent Advances in Noble Metal (Pt, Ru, and Ir)-Based Electrocatalysts for Efficient Hydrogen Evolution Reaction. ACS Omega 2020, 5, 31–40. 10.1021/acsomega.9b03550. 31956748 PMC6963895

[ref7] Liang L.; Zhou H.; Liu B.; Hu C.; Chen D.; Zhu J.; Wang Z.; Li H.-W.; Liu S.; He D.; et al. Ultra-Small Platinum Nanoparticles Segregated by Nickle Sites for Efficient ORR and HER Processes. J. Energy Chem. 2022, 65, 48–54. 10.1016/j.jechem.2021.05.033.

[ref8] Chen P.; Hu X. High-Efficiency Anion Exchange Membrane Water Electrolysis Employing Non-Noble Metal Catalysts. Adv. Energy Mater. 2020, 10, 2002285 10.1002/aenm.202002285.

[ref9] Tiwari J. N.; Harzandi A. M.; Ha M.; Sultan S.; Myung C. W.; Park H. J.; Kim D. Y.; Thangavel P.; Singh A. N.; Sharma P.; et al. High-Performance Hydrogen Evolution by Ru Single Atoms and Nitrided-Ru Nanoparticles Implanted on N-Doped Graphitic Sheet. Adv. Energy Mater. 2019, 9, 1900931 10.1002/aenm.201900931.

[ref10] Jin H.; Sultan S.; Ha M.; Tiwari J. N.; Kim M. G.; Kim K. S. Simple and Scalable Mechanochemical Synthesis of Noble Metal Catalysts with Single Atoms toward Highly Efficient Hydrogen Evolution. Adv. Funct. Mater. 2020, 30, 2000531 10.1002/adfm.202000531.

[ref11] Liu Y.; McCue A. J.; Li D. Metal Phosphides and Sulfides in Heterogeneous Catalysis: Electronic and Geometric Effects. ACS Catal. 2021, 11, 9102–9127. 10.1021/acscatal.1c01718.

[ref12] Li S.; Wang L.; Su H.; Hong A. N.; Wang Y.; Yang H.; Ge L.; Song W.; Liu J.; Ma T.; et al. Electron Redistributed S-Doped Nickel Iron Phosphides Derived from One-Step Phosphatization of MOFs for Significantly Boosting Electrochemical Water Splitting. Adv. Funct. Mater. 2022, 32, 2200733 10.1002/adfm.202200733.

[ref13] You B.; Jiang N.; Sheng M.; Bhushan M. W.; Sun Y. Hierarchically Porous Urchin-Like Ni_2_P Superstructures Supported on Nickel Foam as Efficient Bifunctional Electrocatalysts for Overall Water Splitting. ACS Catal. 2016, 6, 714–721. 10.1021/acscatal.5b02193.

[ref14] Pu Z.; Liu T.; Amiinu I. S.; Cheng R.; Wang P.; Zhang C.; Ji P.; Hu W.; Liu J.; Mu S. Transition-Metal Phosphides: Activity Origin, Energy-Related Electrocatalysis Applications, and Synthetic Strategies. Adv. Funct. Mater. 2020, 30, 2004009 10.1002/adfm.202004009.

[ref15] Owens-Baird B.; Sousa J. P.; Ziouani Y.; Petrovykh D. Y.; Zarkevich N. A.; Johnson D. D.; Kolen’ko Y. V.; Kovnir K. Crystallographic Facet Selective Her Catalysis: Exemplified in FeP and NiP_2_ Single Crystals. Chem. Sci. 2020, 11, 5007–5016. 10.1039/D0SC00676A. 34122957 PMC8159208

[ref16] Schipper D. E.; Zhao Z.; Thirumalai H.; Leitner A. P.; Donaldson S. L.; Kumar A.; Qin F.; Wang Z.; Grabow L. C.; Bao J.; Whitmire K. H. Effects of Catalyst Phase on the Hydrogen Evolution Reaction of Water Splitting: Preparation of Phase-Pure Films of FeP, Fe2P, and Fe3P and Their Relative Catalytic Activities. Chem. Mater. 2018, 30, 3588–3598. 10.1021/acs.chemmater.8b01624.

[ref17] Blanchard P. E. R.; Grosvenor A. P.; Cavell R. G.; Mar A. X-Ray Photoelectron and Absorption Spectroscopy of Metal-Rich Phosphides M_2_P and M_3_P (M = Cr-Ni). Chem. Mater. 2008, 20, 7081–7088. 10.1021/cm802123a.

[ref18] Mu Z.; Guo T.; Fei H.; Mao Y.; Wu Z.; Wang D. Mn-Doped Porous Interconnected MoP Nanosheets for Enhanced Hydrogen Evolution. Appl. Surf. Sci. 2021, 551, 149321 10.1016/j.apsusc.2021.149321.

[ref19] Wang X.; Huang G.; Pan Z.; Kang S.; Ma S.; Shen P. K.; Zhu J. One-Pot Synthesis of Mn_2_P-Mn_2_O_3_ Heterogeneous Nanoparticles in a P, N-Doped Three-Dimensional Porous Carbon Framework as a Highly Efficient Bifunctional Electrocatalyst for Overall Water Splitting. Chem. Eng. J. 2022, 428, 131190 10.1016/j.cej.2021.131190.

[ref20] Chung D. Y.; Jun S. W.; Yoon G.; Kim H.; Yoo J. M.; Lee K.-S.; Kim T.; Shin H.; Sinha A. K.; Kwon S. G.; et al. Large-Scale Synthesis of Carbon-Shell-Coated FeP Nanoparticles for Robust Hydrogen Evolution Reaction Electrocatalyst. J. Am. Chem. Soc. 2017, 139, 6669–6674. 10.1021/jacs.7b01530. 28437070

[ref21] Xiao P.; Chen W.; Wang X. A Review of Phosphide-Based Materials for Electrocatalytic Hydrogen Evolution. Adv. Energy Mater. 2015, 5, 1500985 10.1002/aenm.201500985.

[ref22] Weng C. C.; Ren J. T.; Yuan Z. Y. Transition Metal Phosphide-Based Materials for Efficient Electrochemical Hydrogen Evolution: A Critical Review. ChemSusChem 2020, 13, 3357–3375. 10.1002/cssc.202000416. 32196958

[ref23] Kumar A.; Bui V. Q.; Lee J.; Jadhav A. R.; Hwang Y.; Kim M. G.; Kawazoe Y.; Lee H. Modulating Interfacial Charge Density of NiP_2_-FeP_2_ Via Coupling with Metallic Cu for Accelerating Alkaline Hydrogen Evolution. ACS Energy Lett. 2021, 6, 354–363. 10.1021/acsenergylett.0c02498.

[ref24] Ogundipe T. O.; Shen L.; Shi Y.; Lu Z.; Wang Z.; Tan H.; Yan C. Nickel-Cobalt Phosphide Terephthalic Acid Nano-Heterojunction as Excellent Bifunctional Electrocatalyst for Overall Water Splitting. Electrochim. Acta 2022, 421, 140484 10.1016/j.electacta.2022.140484.

[ref25] Sheng S.; Song Y.; Sha L.; Ye K.; Zhu K.; Gao Y.; Yan J.; Wang G.; Cao D. Simultaneous Hydrogen Evolution and Ethanol Oxidation in Alkaline Medium Via a Self-Supported Bifunctional Electrocatalyst of Ni-Fe Phosphide/Ni Foam. Appl. Surf. Sci. 2021, 561, 150080 10.1016/j.apsusc.2021.150080.

[ref26] Wang M.; Fu W.; Du L.; Wei Y.; Rao P.; Wei L.; Zhao X.; Wang Y.; Sun S. Surface Engineering by Doping Manganese into Cobalt Phosphide Towards Highly Efficient Bifunctional HER and OER Electrocatalysis. Appl. Surf. Sci. 2020, 515, 146059 10.1016/j.apsusc.2020.146059.

[ref27] Chai L.; Liu S.; Pei S.; Wang C. Electrodeposited Amorphous Cobalt-Nickel-Phosphide-Derived Films as Catalysts for Electrochemical Overall Water Splitting. Chem. Eng. J. 2021, 420, 129686 10.1016/j.cej.2021.129686.

[ref28] Lu X. F.; Yu L.; Lou X. W. Highly Crystalline Ni-Doped FeP/Carbon Hollow Nanorods as All-pH Efficient and Durable Hydrogen Evolving Electrocatalysts. Sci. Adv. 2019, 5, eaav6009 10.1126/sciadv.aav6009. 30793034 PMC6377276

[ref29] Xiao X.; Tao L.; Li M.; Lv X.; Huang D.; Jiang X.; Pan H.; Wang M.; Shen Y. Electronic Modulation of Transition Metal Phosphide Via Doping as Efficient and pH-Universal Electrocatalysts for Hydrogen Evolution Reaction. Chem. Sci. 2018, 9, 1970–1975. 10.1039/C7SC04849A. 29675243 PMC5892309

[ref30] Chen Q.; Zhang Q.; Liu H.; Liang J.; Peng W.; Li Y.; Zhang F.; Fan X. Preparation of Hollow Cobalt-Iron Phosphides Nanospheres by Controllable Atom Migration for Enhanced Water Oxidation and Splitting. Small 2021, 17, 2007858 10.1002/smll.202007858. 33690975

[ref31] Arunkumar P.; Gayathri S.; Han J. H. A Complementary Co-Ni Phosphide/Bimetallic Alloy-Interspersed N-Doped Graphene Electrocatalyst for Overall Alkaline Water Splitting. ChemSusChem 2021, 14, 1921–1935. 10.1002/cssc.202100116. 33474804

[ref32] Xu S.; Yu X.; Liu X.; Teng C.; Du Y.; Wu Q. Contrallable Synthesis of Peony-Like Porous Mn-CoP Nanorod Electrocatalyst for Highly Efficient Hydrogen Evolution in Acid and Alkaline. J. Colloid Interface Sci. 2020, 577, 379–387. 10.1016/j.jcis.2020.05.097. 32497919

[ref33] Chen Y.; Wang D.; Meng T.; Xing Z.; Yang X. Modulating the Electronic Structure by Ruthenium Doping Endows Cobalt Phosphide Nanowires with Enhanced Alkaline Hydrogen Evolution Activity. ACS Appl. Energy Mater. 2022, 5, 697–704. 10.1021/acsaem.1c03183.

[ref34] Xu J.; Liu T.; Li J.; Li B.; Liu Y.; Zhang B.; Xiong D.; Amorim I.; Li W.; Liu L. Boosting the Hydrogen Evolution Performance of Ruthenium Clusters through Synergistic Coupling with Cobalt Phosphide. Energy Environ. Sci. 2018, 11, 1819–1827. 10.1039/C7EE03603E.

[ref35] Yang B.; Xu J.; Bin D.; Wang J.; Zhao J.; Liu Y.; Li B.; Fang X.; Liu Y.; Qiao L.; et al. Amorphous Phosphatized Ruthenium-Iron Bimetallic Nanoclusters with Pt-Like Activity for Hydrogen Evolution Reaction. Appl. Catal., B 2021, 283, 119583 10.1016/j.apcatb.2020.119583.

[ref36] Wang X.; Kolen’ko Y. V.; Bao X. Q.; Kovnir K.; Liu L. One-Step Synthesis of Self-Supported Nickel Phosphide Nanosheet Array Cathodes for Efficient Electrocatalytic Hydrogen Generation. Angew. Chem. 2015, 127, 8306–8310. 10.1002/ange.201502577. 26032688

[ref37] Yu L.; Zhang J.; Dang Y.; He J.; Tobin Z.; Kerns P.; Dou Y.; Jiang Y.; He Y.; Suib S. L. In Situ Growth of Ni_2_P-Cu_3_P Bimetallic Phosphide with Bicontinuous Structure on Self-Supported NiCuC Substrate as an Efficient Hydrogen Evolution Reaction Electrocatalyst. ACS Catal. 2019, 9, 6919–6928. 10.1021/acscatal.9b00494.

[ref38] Wu L.; Yu L.; Zhang F.; McElhenny B.; Luo D.; Karim A.; Chen S.; Ren Z. Heterogeneous Bimetallic Phosphide Ni_2_P-Fe_2_P as an Efficient Bifunctional Catalyst for Water/Seawater Splitting. Adv. Funct. Mater. 2021, 31, 2006484 10.1002/adfm.202006484.

[ref39] Morgan D. J. Resolving Ruthenium: XPS Studies of Common Ruthenium Materials. Surf. Interface Anal. 2015, 47, 1072–1079. 10.1002/sia.5852.

[ref40] Lyu Z.; Zhang X. G.; Wang Y.; Liu K.; Qiu C.; Liao X.; Yang W.; Xie Z.; Xie S. Amplified Interfacial Effect in an Atomically Dispersed RuO_x_-on-Pd 2D Inverse Nanocatalyst for High-Performance Oxygen Reduction. Angew. Chem. 2021, 133, 16229–16236. 10.1002/ange.202104013. 33884729

[ref41] Zhao R.; Liu C.; Zhang X.; Zhu X.; Wei P.; Ji L.; Guo Y.; Gao S.; Luo Y.; Wang Z.; Sun X. An Ultrasmall Ru_2_P Nanoparticles-Reduced Graphene Oxide Hybrid: An Efficient Electrocatalyst for NH_3_ Synthesis under Ambient Conditions. J. Mater. Chem. A 2020, 8, 77–81. 10.1039/C9TA10346E.

[ref42] Descostes M.; Mercier F.; Thromat N.; Beaucaire C.; Gautier-Soyer M. Use of XPS in the Determination of Chemical Environment and Oxidation State of Iron and Sulfur Samples: Constitution of a Data Basis in Binding Energies for Fe and S Reference Compounds and Applications to the Evidence of Surface Species of an Oxidized Pyrite in a Carbonate Medium. Appl. Surf. Sci. 2000, 165, 288–302. 10.1016/s0169-4332(00)00443-8.

[ref43] Adroja D. T.; Malik S. Magnetic-Susceptibility and Electrical-Resistivity Measurements on RPdSn (R = Ce-Yb) Compounds. Phys. Rev. B 1992, 45, 779–785. 10.1103/PhysRevB.45.779. 10001118

[ref44] Wang S.; Wu Y.; Cao T.; Wang X.; Long Y.-Z.; Chen D.; Teng B. Magnetic Properties of a Pseudo-One-Dimensional Ferrimagnetic Semiconductor Eu_3_Sb_4_Se_9_. Appl. Phys. Lett. 2020, 117, 232405 10.1063/5.0032920.

[ref45] Jiang Y. C.; Liu G.; Gao J.; Wang J. Anomalous Transition of Major Charge Carriers from Holes to Electrons Observed in Single-Crystal Films of Tungsten. Phys. Rev. B 2016, 94, 245310 10.1103/PhysRevB.94.245310.

[ref46] Xing H.; Wen L.; Shen C.; He J.; Cai X.; Peng J.; Wang S.; Tian M.; Xu Z.-A.; Ku W.; et al. Existence of Electron and Hole Pockets and Partial Gap Opening in the Correlated Semimetal Ca_3_Ru_2_O_7_. Phys. Rev. B 2018, 97, 041113 10.1103/PhysRevB.97.041113.

[ref47] Caputo M.; Khalil L.; Papalazarou E.; Nilforoushan N.; Perfetti L.; Taleb-Ibrahimi A.; Gibson Q.; Cava R. J.; Marsi M. Dynamics of out-of-Equilibrium Electron and Hole Pockets in the Type-II Weyl Semimetal Candidate WTe_2_. Phys. Rev. B 2018, 97, 115115 10.1103/PhysRevB.97.115115.

[ref48] Liang D.; Jiang H.; Xu Q.; Luo J.; Hu Y.; Li C. Modulating the Volmer Step by MOF Derivatives Assembled with Heterogeneous Ni_2_P-CoP Nanocrystals in Alkaline Hydrogen Evolution Reaction. J. Electrochem. Soc. 2018, 165, F1286–F1291. 10.1149/2.0131816jes.

[ref49] Shinagawa T.; Garcia-Esparza A. T.; Takanabe K. Insight on Tafel Slopes from a Microkinetic Analysis of Aqueous Electrocatalysis for Energy Conversion. Sci. Rep. 2015, 5, 13801 10.1038/srep13801. 26348156 PMC4642571

