# Supplementary material for: Ruthenium-Alloyed Iron Phosphide Single Crystal with Increased Fermi Level for Efficient Hydrogen Evolution
Source: ACS Appl Mater Interfaces. 2022 Dec 9;14(50):55587–93. doi: 10.1021/acsami.2c16419 (PMC9782341; doi:10.1021/acsami.2c16419)
Supplement: Supplementary file 1 — am2c16419_si_001.pdf [file am2c16419_si_001.pdf]

Supporting information

Ruthenium-alloyed iron phosphide single crystal with increased Fermi level for efficient hydrogen evolution

Yu Kang[a,#,], Yujia Han[b,#], Hedong Chen[a], Horst Borrmann[a], Peter Adler[a], Darius Pohl[c], Martin Hantusch[d], Markus König[a], Yangkun He[e,*], Yufei Ma[a], Xiaodong Wang[b], Claudia Felser[a,*]*

[a] Max Planck Institute for Chemical Physics of Solids, Nöthnitzer Str. 40, 01187 Dresden, Germany.

[b] Dalian Institute of Chemical Physics, Chinese Academy of Sciences, 457 Zhongshan Road, Dalian, 116023, China.

[c] Dresden Center for Nanoanalysis, cfaed, Technische Universität Dresden, Helmholtzstraße 18, 01069 Dresden, Germany.

[d] Leibniz-Institute for Solid State and Materials Research (IFW), Dresden 01069, Germany.

[e] School of Materials Science and Engineering, Beihang University, Beijing 100191, China.

[#] These authors contribute to the work equally.

[*] Emails: Yu.Kang@cpfs.mpg.de; Claudia.Felser@cpfs.mpg.de; heyangkun@buaa.edu.cn

Experimental details

1. RuFeP powder synthesis

Stoichiometric amount of Ru, Fe, and P powder were uniformly mixed and sealed in a quartz tube with low Ar pressure. The tube was then put into furnace and slowly heated to 800 ℃ holding for 10 days (less than 10 days is also possible). The annealed sample was RuFeP powder.

2. Single crystal growth

The Fe_2P and Ru-Fe_2P single crystals were grown by the Bridgeman method. For Fe_2P, 8 g of 99% Fe_2P powder (Sigma) was put into a MgO crucible, which was sealed in a tantalum tube at Ar atmosphere. The tube was heated to 1450 °C at a rate of 5 ℃ min^{-1} in a vertical Bridgeman furnace to ensure the melting of the powder, then the sample was moved down slowly at a rate of 0.011 mm/min to crystallize the liquid. After holding for 120 h, the furnace was cooled to 400 ℃ at a rate of 5 ℃/min, and then naturally cooled to room temperature. The obtained sample ingot was cut with the bottom part as Fe_2P single crystal. For Ru-Fe_2P growth, the suitable amount of RuFeP and Fe_2P powders were stoichiometrically mixed. The single crystal growth was the same as that of Fe_2P. The obtained single crystal was orientated by Laue diffraction instrument and cut with wire saw.

3. Characterizations

The single crystals orientation was identified by the Laue diffraction instrument. High-resolution transmission electron microscopy (HRTEM) and energy dispersive spectroscopy (EDS) were performed by a JEOL F200 with an operation voltage of 80-200 kV. Prior to the tests, samples were thinned using a focused ion beam (FIB) technique with ion acceleration voltages as low as 5 kV. X-ray photoelectron spectroscopy (XPS) was conducted on a PHI 5600 spectrometer. An Al Kα X-ray source (200 W) was used at a pass energy of 11.95 eV. Hall transport and resistivity measurements were carried out on a Quantum Design PPMS. ^{57}Fe

Mössbauer spectra were collected at 6 K with a standard WissEl spectrometer which was operated in the constant acceleration mode with a $^{57}Co/Rh$ source. About 15 mg of Fe_2P or Ru-Fe_2P powder were mixed with BN and distributed in an acrylic glass sample container (inner diameter 13mm). The latter was placed in Janis-SHI 850-5 closed cycle refrigerator. The spectra were analyzed using the MossWinn software.[1] Isomer shifts were referred to alpha-iron.

4. Electrochemical measurements

The single crystal was polished first and attached with Ti wire by silver paint. The crystal was covered by resin to expose specific surface. The electrocatalytic properties were measured on an Autolab PGSTAT302N electrochemistry workstation with a three-electrode system. Ag/AgCl electrode and carbon rod were used as the reference electrode and counter electrode, respectively. All the electrochemical measurements were carried out in 1 M KOH solution. Linear Sweep Voltammetry (LSV) was tested at a sweep rate of 2 mV/s and compensated by 90% iR correction. Electrochemical impedance spectroscopy was conducted from 50 kHz to 0.1 Hz. The applied potential against reference electrode is converted into potential versus RHE by

$$E_{vs.RHE} = E_{vs.Ag/AgCl} + 0.197 + 0.059 \times pH. \quad (S1)$$

Where the pH of 1 M KOH in this work measured by HI 9125 pH meter was 13.80.

To calculate the turnover frequency (TOF), the following equation is used:

$$TOF = \frac{number\ of\ H_2\ turnovers}{number\ of\ active\ sites} \quad (S2)$$

The number of H_2 turnovers is expressed as:

$$No.\ of\ H_2 = (|j|\frac{mA}{cm^2})(\frac{1\ C\ s^{-1}}{1000\ mA})(\frac{1\ mol\ e^{-1}}{96485.3\ C})(\frac{1\ mol\ H_2}{2\ mol\ e^{-1}})(\frac{6.022 \times 10^{23}\ H_2\ moleculars}{1\ mol\ H_2})$$

$$= 3.12 \times 10^{15} \frac{H_2\ s^{-1}}{cm^2} \times |j| \quad (S3)$$

Here there are two assumptions: 1) 9 atoms are exposed per unit cell and are regarded as the active sites. 2) The number of atoms per cm^2 of $(2\bar{1}1)$ plane is the same as that of (001). Therefore, the surface area of the one unit cell for (001) plane is obtained by:

$$S = \frac{\sqrt{3}}{2}a^2 = 29.7755\ A^2 \tag{S4}$$

The real surface area is considered as the exposed geometric surface area for the single crystals. Hence, the number of atoms per cm^2 is expressed by:

$$\text{No. of atoms } per\ cm^2 = 9 \times \frac{1\ cm^2}{29.7755\ A^2} = 3.023 \times 10^{15} cm^{-2} \tag{S5}$$

Finally, the TOF is calculated by:

$$TOF = \frac{number\ of\ H_2\ turnovers}{number\ of\ active\ sites} = \frac{3.12 \times 10^{15} \frac{H_2\ s^{-1}}{cm^2}}{3.023 \times 10^{15} cm^{-2}} \times |j| = 1.032|j|\ H_2/s \tag{S6}$$

5. Computational details

In this study, all first-principle calculations are performed by the density functional theory (DFT) using the Vienna Ab-initio Simulation Package (VASP) code.[2] The generalized gradient approximation (GGA) with the exchange-correlation functional Perdew-Burke-Ernzerhof (PBE) is adopted to describe the electronic exchange and correlation effects.[3-5] The cutoff energy for the plane-wave basis is set to 600 eV in all the cases. The convergence of geometric optimization is checked with the forces less than 0.03 eV Å^{-1} and the energy difference less than 10^{-6} eV. For geometry optimization, bulk electronic structure calculations and surface geometry optimizations, their Brillouin-zone integrations are performed using 7×7×10, 10×10×14 and 3×3×1 Monkhorst-Pack mesh,[6] respectively. In order to investigate the effect of Ru incorporation on the electronic properties, we replace the Fe atom with one Ru atom at the pyramidal sites in the unit-cell according to the experimental results. To study the catalytic reactivity of Fe_2P-001, Ru-Fe_2P-001 and Ru-Fe_2P-$2\bar{1}1$, a series of surface models of 2×2

supercells are constructed, as shown in Figure S4, 5. The surface of Ru-Fe_2P-001 and Ru-Fe_2P-2$\bar{1}$1 are constructed by replacing one Fe atom with Ru at the pyramidal site of Fe_2P surface. A 15 Å vacuum layer is applied to separate the periodic model in the z-direction of the slab models. The D3 Grimme correction is adopted for the consideration of van der Waals (vdW) interactions in all reaction processes.[7] Each slab has two layers of repeating units. The upper one and adsorbates are relaxed, while the remaining bottom one is kept fixed to simulate the bulk during the structural relaxation. The nudged elastic band (NEB) combined with minimum-mode following dimer method is employed to obtain the transition state structure of the reaction.[8] Moreover, the calculations are considered converged when the maximum forces on all atoms are less than 0.05 eV Å^{-1}. Meanwhile, the transition state is verified using the single imaginary frequency. All structure visualizations are supported by VESTA3 software.[9]

The free energies (ΔG_{ads}) of H_2O adsorption on supports are estimated by the following formula:

$$\Delta G_{ads} = G_{H2O^* + support} - G_{support} - G_{H2O(l)} \quad \text{(S7)}$$

where $G_{H2O^*+support}$ is the total energy of the supports decorated with H_2O molecule, $G_{support}$ is the total energy of supports, and $G_{H2O(l)}$ is the total energy of H_2O(l) molecule, which is equal to the energy of water vapor at saturated vapor pressure.

The free energies (ΔG_a) of H_2O activation on supports are estimated by the following formula:

$$\Delta G_a = G_{TS} - G_{H2O^*+ support} \quad \text{(S8)}$$

where G_{TS} is the total energy of transition states of H_2O dissociated on supports.

The free energies ($\Delta G_{des,\,H}$) of intermediate H* desorption from supports are estimated by the following formula:

$$\Delta G_{des,\,H} = G_{H2(g)}/2 + G_{support} - G_{H^*} \quad \text{(S9)}$$

where $G_{H2(g)}$ is the total energy of H_2(g) molecule and G_{H*} is the total energy of the supports decorated with H atom.

The free energies ($\Delta G_{des,\ OH}$) of intermediate OH* desorption on supports is estimated by the following formula:

$$\Delta G_{des,\ OH} = G_{H*} + G_{OH-} - G_{H*\ +\ OH*} - G_{e-} \qquad \text{(S10)}$$

where $G_{H*\ +\ OH*}$ is the total energy of the supports decorated with H and OH species, G_{e-} is the energy of electron, G_{OH-} is the total energy of OH^-, which can be calculated by the difference between $G_{H2O(l)}$ and G_{H+}.

All Gibbs free energy calculations are performed at 298.15 K using vaspkit code.[10]

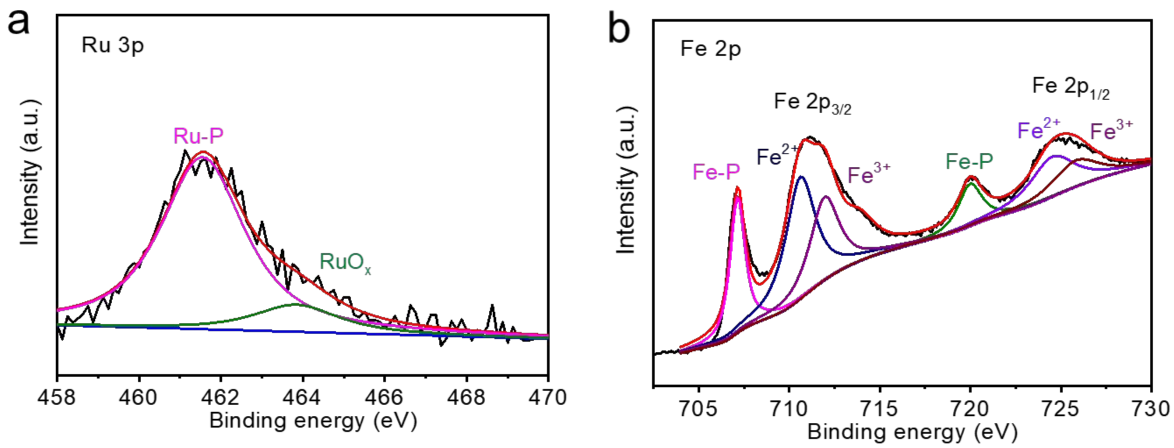

Figure S1. XPS results of (a) Ru 3p and (b) Fe 2p spectra for Ru-Fe_2P single crystal.

Table S1. The occupation states of atoms derived from the single crystal XRD refinement.

**Atom**	**WP**[a]	**x**	**y**	**z**	$\mathbf{U_{eq}}$	**Occ.**
Fe1	3f	0	0.40530(9)	0	0.00833(12)	0.938(7)
Ru1	3f	0	0.40530(9)	0	0.00833(12)	0.062(7)
Fe2	3g	0.25708(8)	0.25708(8)	0.5	0.00643(10)	0.997(6)
P1	1a	0	0	0	0.0073(2)	0.999(11)
P2	2d	0.333333	0.666667	0.5	0.00683(15)	1.005(7)

[a] Wyckoff position.

Table S2. Crystal data and parameters of the refinement.

$(Fe_{0.97}Ru_{0.03})_2P$	D_x = 6.999 Mg m^{-3}
Mr = 145.38	Mo Kα radiation, λ = 0.71073 Å
Hexagonal, $P\bar{6}2m$	Cell parameters from 1126 reflections, 1610
a = 5.8605 (3) Å	θ = 4–38.3°, 12.2–43.7°
c = 3.4842 (2) Å	μ = 21.45 mm^{-1}
V = 103.63 (1) $Å^3$	T = 295 K
Z = 3	Prism, grey
F (000) = 204	0.12 × 0.09 × 0.04 mm^3
$wR(F^2)$ = 0.036	$R[F^2 > 2\sigma(F^2)]$ = 0.015

Pyramidal site	Geometric parameters (Å)	Tetrahedral site	Geometric parameters (Å)
Fe1—P1	2.3753 (6)	Fe2—P2	2.2110 (3)
Fe1—P2	2.4910 (1)	Fe2—$P2^{viii}$	2.2110 (3)
Fe1—$P2^{i}$	2.4910 (1)	Fe2—P1	2.3032 (3)
Fe1—$P2^{ii}$	2.4910 (1)	Fe2—$P1^{ix}$	2.3032 (3)
Fe1—$P2^{iii}$	2.4910 (1)		

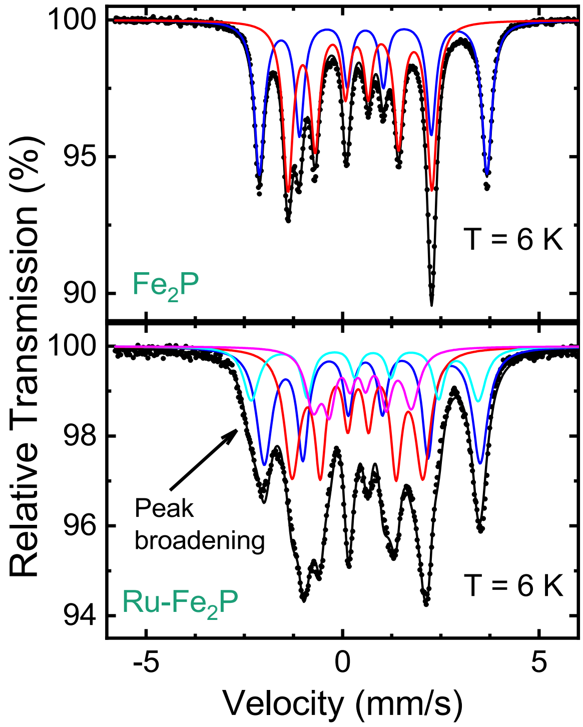

Figure S2. Mössbauer spectra of Fe_2P and Ru-Fe_2P in the magnetically ordered phase at the temperature of 6 K. In agreement with earlier results,[11] the spectra of Fe_2P exhibit two magnetic hyperfine sextets which are assigned to the two crystallographically inequivalent Fe1 (isomer shift IS = 0.68 mm/s, hyperfine field B_{hf} = 18.00 T) and Fe2 (IS = 0.40 mm/s, B_{hf} = 11.39 T) sites. The larger B_{hf} for the square pyramidal Fe1 site reflects the larger ordered magnetic moments. It is apparent that the hyperfine pattern of Ru-Fe_2P is strongly broadened owing to the fact that substitution of the magnetic Fe by non-magnetic Ru atoms disturbs the dominant Fe1-Fe1 and Fe1-Fe2 exchange pathways which determine the magnetic structure. We have fitted the spectrum by four sextet components with Gaussian B_{hf} distributions as indicated by the colored subspectra. Based on the isomer shifts the outer components are assigned to Fe1 (IS = 0.68 mm/s) and the inner components to Fe2 (IS = 0.38 mm/s). Notably, a broad Fe2 component with considerably reduced average B_{hf} gives rise to enhanced intensity in the center of the spectrum. This may correspond to Fe2 sites with a Ru atom in their environment.

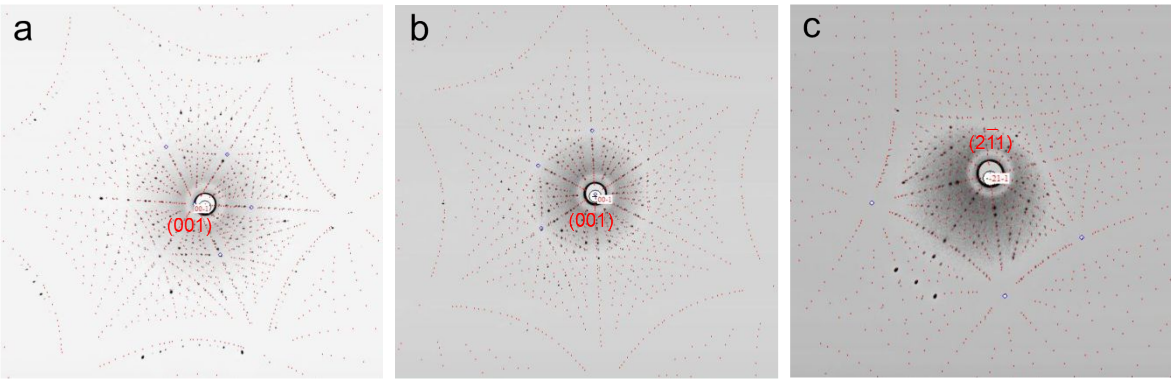

Figure S3. Laue diffraction patterns of (a) Fe_2P-001, (b) Ru-Fe_2P-001, and (c) Ru-Fe_2P-$2\bar{1}1$. The black and red dot patterns are experimental and simulated results, respectively.

Table S3. Table of comparison of metal phosphides for HER.

Catalysts	Electrolyte	Overpotential mV	Tafel slope mV/dec	C_{dl} mF/cm^2	Reference
Ru-Fe_2P-211	1 KOH	318	86	0.35	This work
CoP	1 KOH	190	143	2.1	12
Ni_2P-Cu_3P	1 KOH	102	183	46.3	13
CCS-NiFeP-20	1 KOH	56	38	31.9	14
FeP-[010]	0.5 H_2SO_4	209	-	0.039	15
$Ni_2P/NiTe_2$	1 KOH	62	80	13.9	16
FePN/CNT-200	1 M PBS	158	87	14.5	17
CoNiP/CoNi/N RGO	1 KOH	150	97	0.015	18
NiCoFeP/C	1 KOH	149	108	97.2	19
Ru–MnFeP/NF	0.5 H_2SO_4	38	46	-	20
Co-Co_2P@NPC/rGO	0.5 H_2SO_4	136	50.6	9.99	21
$(Fe_{0.14}Ni_{0.47}Co_{0.39})_2P$/ CFP	1 KOH	95	98	15.81	22
NiCoP–CoP/NF	1 KOH	73	91.3	120.9	23
$Ni_{0.25}Rh_{1.75}P$	1 KOH	82.1	63	12.7	24

Table S4. Electrochemical impedance spectra fitting parameters in Fig. 3f.

Element	Parameter	Sample		
		Fe_2P-001	Ru-Fe_2P-001	Ru-Fe_2P-$2\bar{1}\bar{1}$
R_s	R(Ω)	7.33	7.77	7.57
R_{p1}	R(Ω)	2270	175	107
CPE1	Y_0(μMho*s^N)	66.5	43.5	46.9
	N	0.804	0.8	0.819

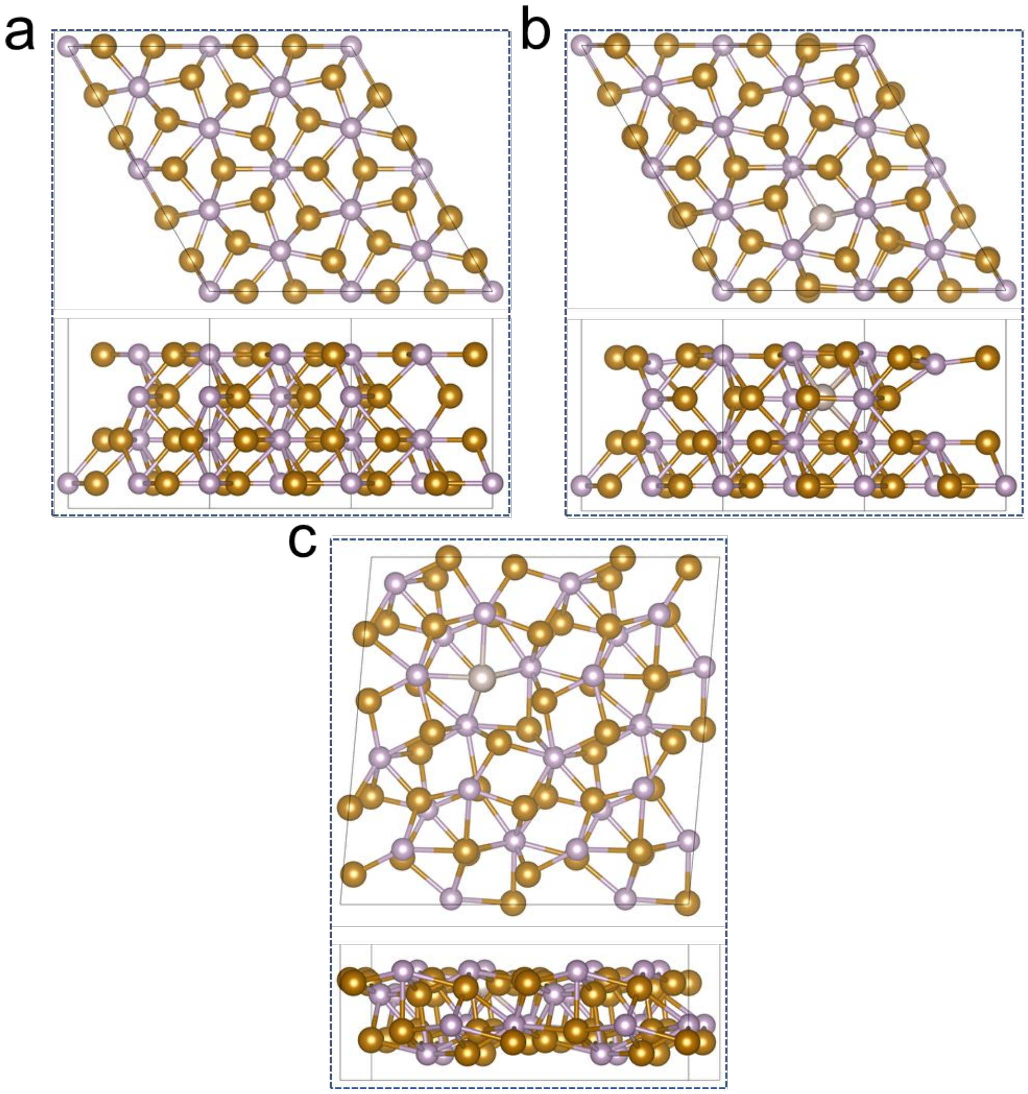

Figure S4. Top (up) and side (down) view of optimized (a) Fe_2P-001, (b) Ru-Fe_2P-001 and (c) Ru-Fe_2P-$2\bar{1}1$surface. The brown, purple and gray balls represent the Fe, P and Ru atoms, respectively.

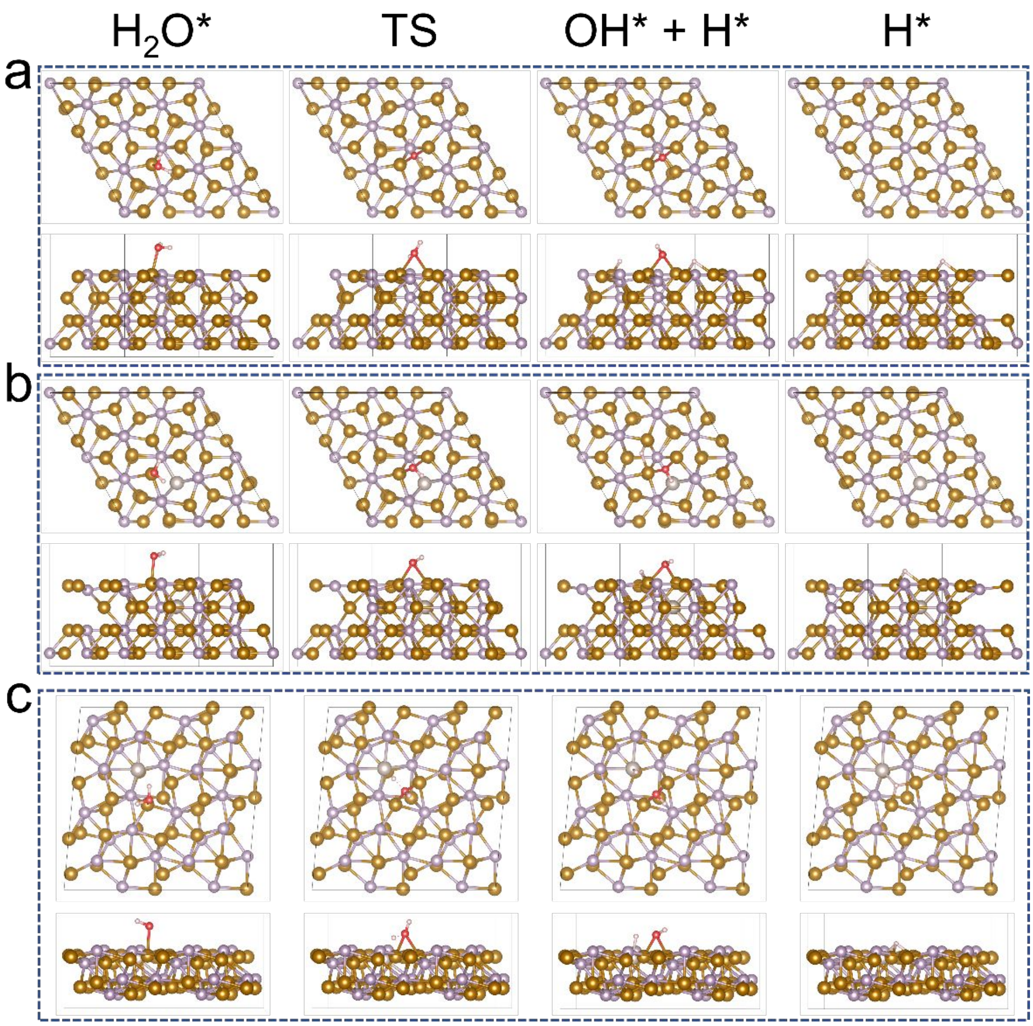

Figure S5. Top (up) and side (down) view of states of water dissociation on (a) Fe_2P-001, (b) Ru-Fe_2P-001 and (c) Ru-Fe_2P-$2\bar{1}1$ surface. The brown, purple, gray, red and white balls represent the Fe, P, Ru, O and H atoms, respectively.

References:

(1) Klencsár, Z.; Kuzmann, E.; Vértes, A. User-Friendly Software for Mössbauer Spectrum Analysis. *J. Radioanal. Nucl. Chem.* **1996**, *210*, 105-118.

(2) Kresse, G.; Furthmüller, J. Efficiency of Ab-Initio Total Energy Calculations for Metals and Semiconductors Using a Plane-Wave Basis Set. *Comput. Mater. Sci.* **1996**, *6*, 15.

(3) Blöchl, P. E. Projector Augmented-Wave Method. *Phys. Rev. B* **1994**, *50*, 17953.

(4) Perdew, J. P.; Burke, K.; Ernzerhof, M. Generalized Gradient Approximation Made Simple. *Phys. Rev. Lett.* **1996**, *77*, 3865.

(5) Kresse, G.; Joubert, D. From Ultrasoft Pseudopotentials to the Projector Augmented-Wave Method. *Phys. Rev. B* **1999**, *59*, 1758.

(6) Monkhorst, H. J.; Pack, J. D. Special Points for Brillouin-Zone Integrations. *Phys. Rev. B* **1976**, *13*, 5188.

(7) Grimme, S.; Antony, J.; Ehrlich, S.; Krieg, H. A Consistent and Accurate Ab Initio Parametrization of Density Functional Dispersion Correction (DFT-D) for the 94 Elements H-Pu. *J. Chem. Phys.* **2010**, *132*, 154104.

(8) Henkelman, G. A Climbing Image Nudged Elastic Band Method for Finding Saddle Points and Minimum Energy Paths. *J. Chem. Phys.* **2000**, *113*, 9901.

(9) Momma, K.; Izumi, F. VESTA3 for Three-Dimensional Visualization of Crystal, Volumetric and Morphology Data. *J. Appl. Crystallogr.* **2011**, *44*, 1272.

(10) Wang, V.; Xu, N.; Liu, J.-C.; Tang, G.; Geng, W.-T. Vaspkit: A User-Friendly Interface Facilitating High-Throughput Computing and Analysis Using VASP Code. *Comput. Phys. Commun.* **2021**, *267*, 108033.

(11) Kobayashi, H.; Umemura, J.; Zhang, X.-W.; Uwatoko, Y. Magnetic Properties of Fe_2P Single-Crystal under Multi-Extreme Conditions. *J. Phys.: Conf. Ser.* **2008**, *121*, 032009.

(12) Chen, Y.; Wang, D.; Meng, T.; Xing, Z.; Yang, X. Modulating the Electronic Structure by Ruthenium Doping Endows Cobalt Phosphide Nanowires with Enhanced Alkaline Hydrogen Evolution Activity. *ACS Appl. Energy Mater.* **2022**, *5*, 697-704.

(13) Yu, L.; Zhang, J.; Dang, Y.; He, J.; Tobin, Z.; Kerns, P.; Dou, Y.; Jiang, Y.; He, Y.; Suib, S. L. In Situ Growth of Ni_2P-Cu_3P Bimetallic Phosphide with Bicontinuous Structure on Self-Supported NiCuC Substrate as an Efficient Hydrogen Evolution Reaction Electrocatalyst. *ACS Catal.* **2019**, *9*, 6919-6928.

(14) Li, S.; Wang, L.; Su, H.; Hong, A. N.; Wang, Y.; Yang, H.; Ge, L.; Song, W.; Liu, J.; Ma, T. Electron Redistributed S-Doped Nickel Iron Phosphides Derived from One-Step Phosphatization of MOFs for Significantly Boosting Electrochemical Water Splitting. *Adv. Funct. Mater.* **2022**, *32*, 2200733.

(15) Owens-Baird, B.; Sousa, J. P.; Ziouani, Y.; Petrovykh, D. Y.; Zarkevich, N. A.; Johnson, D. D.; Kolen'ko, Y. V.; Kovnir, K. Crystallographic Facet Selective HER Catalysis: Exemplified in FeP and NiP_2 Single Crystals. *Chem. Sci.* **2020**, *11*, 5007-5016.
(16) Li, Y.; Tan, X.; Tan, H.; Ren, H.; Chen, S.; Yang, W.; Smith, S. C.; Zhao, C. Phosphine Vapor-Assisted Construction of Heterostructured $Ni_2P/NiTe_2$ Catalysts for Efficient Hydrogen Evolution. *Energy Environ. Sci.* **2020**, *13*, 1799-1807.
(17) Zhao, S.; Xie, R.; Kang, L.; Yang, M.; He, X.; Li, W.; Wang, R.; Brett, D. J.; He, G.; Chai, G. Enhancing Hydrogen Evolution Electrocatalytic Performance in Neutral Media Via Nitrogen and Iron Phosphide Interactions. *Small Science* **2021**, *1*, 2100032.
(18) Arunkumar, P.; Gayathri, S.; Han, J. H. A Complementary Co-Ni Phosphide/Bimetallic Alloy-Interspersed N-Doped Graphene Electrocatalyst for Overall Alkaline Water Splitting. *ChemSusChem* **2021**, *14*, 1921-1935.
(19) Wei, X.; Zhang, Y.; He, H.; Peng, L.; Xiao, S.; Yao, S.; Xiao, P. Carbon-Incorporated Porous Honeycomb NiCoFe Phosphide Nanospheres Derived from a MOF Precursor for Overall Water Splitting. *Chem. Commun.* **2019**, *55*, 10896-10899.
(20) Cai, J.; Song, Y.; Zang, Y.; Niu, S.; Wu, Y.; Xie, Y.; Zheng, X.; Liu, Y.; Lin, Y.; Liu, X. N-Induced Lattice Contraction Generally Boosts the Hydrogen Evolution Catalysis of P-Rich Metal Phosphides. *Sci. Adv.* **2020**, *6*, eaaw8113.
(21) Li, G.; Yu, J.; Jia, J.; Yang, L.; Zhao, L.; Zhou, W.; Liu, H. Cobalt-Cobalt Phosphide Nanoparticles@ Nitrogen-Phosphorus Doped Carbon/Graphene Derived from Cobalt Ions Adsorbed Saccharomycete Yeasts as an Efficient, Stable, and Large-Current-Density Electrode for Hydrogen Evolution Reactions. *Adv. Funct. Mater.* **2018**, *28*, 1801332.
(22) Pang, L.; Liu, W.; Zhao, X.; Zhou, M.; Qin, J.; Yang, J. Engineering Electronic Structures of Nickel Cobalt Phosphide Via Iron Doping for Efficient Overall Water Splitting. *ChemElectroChem* **2020**, *7*, 4913-4921.
(23) Liu, H.; Ma, X.; Hu, H.; Pan, Y.; Zhao, W.; Liu, J.; Zhao, X.; Wang, J.; Yang, Z.; Zhao, Q. Robust NiCoP/CoP Heterostructures for Highly Efficient Hydrogen Evolution Electrocatalysis in Alkaline Solution. *ACS Appl. Mat. Interfaces* **2019**, *11*, 15528-15536.
(24) Batugedara, T. N.; Brock, S. L. Role of Noble-and Base-Metal Speciation and Surface Segregation in $Ni_{2-x}Rh_xP$ Nanocrystals on Electrocatalytic Water Splitting Reactions in Alkaline Media. *Chem. Mater.* **2022**, *34*, 4414-4427.
